# Depression and Associated Factors Among Older Adults in a North Indian State: A Cross-Sectional Study

**DOI:** 10.7759/cureus.35962

**Published:** 2023-03-09

**Authors:** Aninda Debnath, Chirag Sandooja, Jugal Kishore

**Affiliations:** 1 Department of Community Medicine, Vardhman Mahavir Medical College and Safdarjung Hospital, New Delhi, IND

**Keywords:** elderly population, geriatric psychiatry, patient health questionnaire (phq-9), depression, mental health, mental health services

## Abstract

Introduction: India is currently experiencing a significant increase in its elderly population, and it is predicted to rise. Depression is prevalent among the elderly population. This study aimed at measuring the prevalence of depression among the elderly population in India.

Methodology: This cross-sectional study was conducted in both urban and rural regions of Delhi, with a total of 230 participants recruited through systematic random sampling. This sampling method involved selecting households from a comprehensive list. The Patient Health Questionnaire 9 (PHQ-9) was used as a screening tool for depression. Participants with a PHQ-9 score above 9 were considered to potentially experience depression.

Results: The study findings revealed that 68.2% (95%CI: 61.8%-74.2%) of the total sample of 230 participants screened positive for depression. Gender (*p* = 0.02), age category (*p* < 0.01), place of residence (*p* < 0.01), and diabetes (*p* < 0.01) were significantly associated with depression.

Conclusion: The study found a high prevalence of depression among the elderly population, with females, urban dwellers, and those with a history of diabetes being significantly associated with depression. Early detection through screening programs and community-based interventions could help manage depression in this vulnerable group.

## Introduction

India is facing a rapidly growing elderly population, with life expectancy having increased over the past 70 years [1]. As of 2021, it is estimated that around 120 million individuals in India, or approximately 8% of the total population, are aged 60 or older. This trend is projected to continue, with predictions suggesting that by 2050, there will be around 340 million elderly individuals in India [2]. This increase in the elderly population presents several challenges, including increased demand for healthcare services, elevated risk of chronic diseases, and decreased workforce participation [3,4]. It also underscores the need for policies and interventions aimed at improving the well-being and quality of life of the elderly population in India [5]. The aging process can negatively impact mental health, as the elderly must not only deal with physical aging but also address challenges affecting their mental and social well-being. Factors such as disability from illness, loneliness, lack of family support, limited personal autonomy, and financial dependency can contribute to an increase in mental health issues, particularly depression [6-8]. A recent systematic review and meta-analysis showed that the prevalence of depression among the elderly population in India was 34.4% (95%CI: 29.3-39.7) [9]. However, the coronavirus disease 2019 (COVID-19) pandemic has exacerbated this condition. The pandemic has created an environment where many determinants of poor mental health have been exacerbated, leading to an additional 10.7 million (95%CI: 7.21-14.9) disability-adjusted life years (DALYs) for major depressive disorder globally. The Southeast Asia region has seen a 36% increase in major depressive symptoms, including among the elderly population [10]. Depression can greatly impact the quality of life and increase dependence on others, and if left untreated, can have serious clinical and social implications. Early recognition, diagnosis, and intervention, such as treatment initiation and rehabilitation, can prevent suffering, and premature death, and lead to a productive and autonomous life. Early diagnosis and intervention can also significantly reduce mortality rates due to suicide and medical illnesses, as well as lower healthcare costs [9,10]. The COVID-19 pandemic has had a significant impact on mental health globally, and the elderly population is particularly vulnerable. With India's rapidly growing elderly population and increased life expectancy, the well-being of this demographic is of paramount importance. This study aimed at measuring the prevalence of depression among the elderly population in India and providing important insights into the current state of mental health in this demographic.

## Materials and methods

Study settings

The present cross-sectional study was conducted between May 2021 and August 2021, in two locations, Aliganj and Fatehpur Beri, in the South Delhi district of New Delhi, India. Aliganj, being an urbanized village, has a population of 6000 individuals, while Fatehpur Beri, a rural village, serves a larger community of 57,000 individuals.

Study population and sampling strategy

The study participants were elderly individuals (aged 60 years and above) residing in the designated study areas, fulfilling the inclusion criteria of being residents of the area for at least six months. Efforts were made to contact all eligible participants, and those who could not be reached even after three attempts were excluded from the study. The sample size was calculated based on the prevalence of 34.4%, with a desired relative precision of 20%, a 95% confidence level, and an expected 10% non-response rate, resulting in a minimum sample size of 230 [9]. A complete list of households with elderly individuals was obtained, and systematic random sampling was used to select 115 participants from each study area, making a total of 230 participants. The first house to be included in the study was determined through simple random sampling, and then every nth (n=7) house was selected thereafter. Those who were aged more than 60 years were included in the study. Participants with medical conditions that can affect mood such as dementia, Parkinson's disease, or stroke, and Individuals with severe psychiatric disorders such as schizophrenia and bipolar disorder were excluded from the study.

Study instrument

A comprehensive literature review and expert consultation were employed to design a semi-structured interview schedule aimed at capturing the participants' perceptions and experiences. The schedule incorporated socio-demographic characteristics, such as the participants' education and work history, as well as details related to their communication with family members and personal information relevant to depression, including substance use, presence of chronic illnesses (such as hypertension and diabetes), and sleeping habits. Individuals who adhered to a consistent sleep schedule characterized by regular sleep onset and offset times were defined as having regular sleeping habits. Conversely, individuals who experienced varying sleep onset and offset times on a daily basis were considered to have irregular sleeping habits. The study defined chronic illness as an ailment lasting for three months or more, or an illness with recurrent occurrences. Adequate physical activity was defined as a minimum of 30 minutes of moderate activity on a daily basis. The interview schedule underwent pre-testing and was subsequently refined before being administered in the local language.

The Patient Health Questionnaire (nine-item version) (PHQ-9) was used to screen for depression in this study. The PHQ-9 is a short scale comprising nine questions, each of which pertains to a symptom of depression, enabling a criteria-based diagnosis. The score for each item ranges from zero (not at all) to three (nearly every day), with a minimum and maximum score of zero and 27, respectively. Higher scores indicate greater severity of depression. A cut-off score of nine has been found to be 88% sensitive and 80% specific for depression among elderly populations [11]. The interviewer, a medical graduate, was trained in the administration of the study tools by an experienced psychiatrist in the psychiatry outpatient clinic of Vardhman Mahavir Medical College and Safdarjung Hospital, New Delhi.

Study process

Data collection was conducted through house-to-house visits. If a participant was not present during the first visit, a subsequent visit was scheduled after confirming their availability with family members or neighbors. Participants who could not be located after three visits were recorded as non-respondents. The semi-structured interview schedule, which incorporated the PHQ-9, was administered as per protocol. Participants who scored nine or higher on the PHQ-9 were considered positive for depression.

Statistical analysis

Statistical analysis was performed using Stata Statistical Software, release 17. The prevalence of depression was expressed as a percentage with 95% confidence intervals (CI). The relationship between depression and variables in the interview schedule was assessed using univariate and multivariate logistic regression analysis. Variables were selected for entry into the multivariable regression model based on a cut-off value of p = 0.25. Unadjusted and adjusted odds ratios (OR) and 95% CIs were calculated, and results were considered statistically significant if the p-value was less than 0.05.

Ethical clearance

Ethical clearance was obtained from the Institutional Ethics Committee of Vardhman Mahavir Medical College and Safdarjung Hospital, New Delhi (approval number: IEC/VMMC/SJH/Thesis/2021/CC-77). Each subject enrolled in the study was explicitly explained the purpose of the study by the investigator and informed written consent was obtained, prior to inclusion.

## Results

Our study included 230 patients aged more than 60 years, 115 each from Aliganj and Fatehpur Beri. In our study, 52.1% of the participants were females (n=120). The average age of the participants was 66.8 years (SD 6.5), with most participants in the age group of 60-69 years, and 61.3% were married. The percentage of unmarried, divorced, or widowed participants was more among females compared to males. Half of the participants (50.4%) were either illiterate or just literate. Of our participants, 6.5% were living alone, 20.9% were living in joint families, and most (72.6%) were living in nuclear families. Among all the participants, only 16.9% (n=39) had any life insurance. There were significant differences between age categories, marital status, education status, diet preferences, and possession of health insurance among genders (Table 1).

**Table 1:** Sociodemographic characteristics of the participants (N =230) * = Statistically significant, Test of signifiance = Chi-square test

Variables	Gender		p-value
	Male (n=110) n (%)	Female (n=120) n (%)	
Age category			
60 - 69 Years	74 (47.4)	82 (52.6)	<0.01*
70-79 Years	34 (58.6)	24 (41.4)	
More than 80 years	2 (12.5)	14 (87.5)	
Marital Status			
Married	88 (62.4)	53 (37.6)	<0.01*
Unmarried/Divorced/Widowed	22 (24.7)	67 (75.3)	
Religion			
Hindu	92 (46.7)	105 (53.3)	0.40
Muslim/Sikh/Christian	18 (54.5)	15 (45.5)	
Education			
Illiterate or just literate	26 (22.4)	90 (77.6)	<0.01*
Educated up to intermediate	60 (68.9)	27 (31.1)	
Educated up to graduated or more	24 (88.9)	39 (11.1)	
Type of family			
Living alone	8 (53.3)	7 (46.7)	0.9
Nuclear family	79 (47.3)	88 (52.7)	
Joint family	23 (47.9)	25 (52.1)	
Diet Preference			
Vegetarian	43 (38.1)	70 (61.9)	<0.01*
Non-vegetarian	67 (57.3)	50 (42.7)	
Any Health Insurance			
Have health insurance	28 (71.8)	11 (28.2)	<0.01*
No health insurance	82 (42.9)	109 (57.1)	
Urban/Rural			
Urban	47 (40.9)	68 (59.1)	0.03
Rural	63 (54.8)	52 (45.2)	

**Figure 1:**
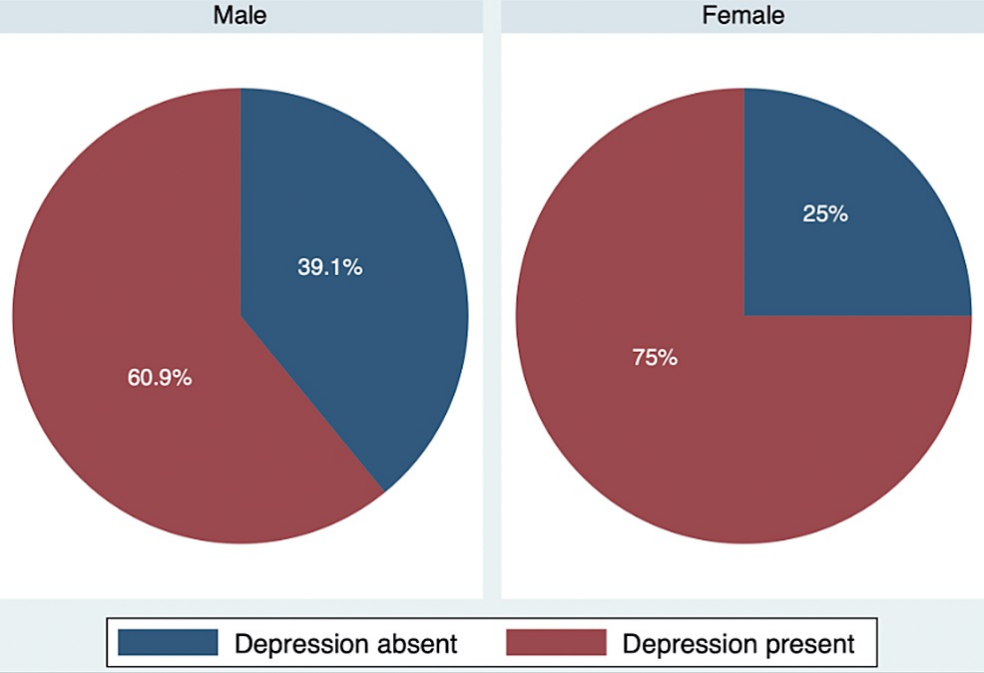
Prevalence of depression by gender

The results of the study showed that out of 230 participants, 68.2% (95%CI: 61.8% - 74.2%) of the participants screened positive for depression (PHQ score more than 9). The prevalence of depression was more in females, 75% (95%CI: 66.2% - 82.4%]). In male participants, the prevalence was 60.9% (95%CI: 51.1%-70.1%) (Figure 1).

As demonstrated in Table 2, a statistically significant association was observed between depression and sex, with a higher prevalence of depression among females compared to males (75% versus 60.9%, p = 0.02). The prevalence of depression was found to be higher among participants aged less than 80 years compared to other age groups. However, the association between depression and age category, marital status, and education status was not statistically significant. Regarding the location of residence, those living in urban areas were more likely to suffer from depression compared to those in rural areas, with the prevalence of depression being 76.5% and 60%, respectively (p < 0.01). The prevalence of depression was subsequently found to be higher among those living alone, in nuclear families, and in joint families, with rates of 73.3%, 67.1%, and 70.8%, respectively. No significant association was found between depression and type of family, diet preference, or the presence of health insurance. However, participants with hypertension and diabetes had a higher prevalence of depression compared to those without these conditions. Specifically, the prevalence of depression was 75% among hypertensives compared to 59.8% among non-hypertensives (p < 0.01), and 80.5% among diabetics compared to 54.2% among non-diabetics (p < 0.01). The prevalence of depression was also higher among smokers compared to non-smokers (77.4% versus 65.5%, p=0.10). However, the association between depression and exercise was not statistically significant. In terms of sleeping habits, participants with irregular sleeping patterns had a higher prevalence of depression compared to those with regular sleeping patterns (74.3% versus 57.3%, p < 0.01). Additionally, participants with an Epworth Sleepiness Scale score of less than 9 had a lower prevalence of depression compared to those with higher scores.

**Table 2:** Association of depression status and participant characteristics * = Statistically significant; test of significance = Chi-square test

Variables	Depression (n= 157), n (%)	No depression (n= 73), n (%)	p-value
Gender			
Male	67 (60.9%)	43 (39.1%)	0.02*
Female	90 (75%)	30 (25%)	
Age Category			
60-69 years	109 (69.9%)	47 (30.1%)	0.26
70-79 years	40 (68.9%)	18 (31.1%)	
More than 80 years	8 (50%)	8 (50%)	
Marital Status			
Married	95 (67.4%)	46 (32.6%)	0.71
Unmarried/Divorced/Widowed	62 (69.7%)	27 (30.3%)	
Religion			
Hindu	138 (70.1%)	59 (29.9%)	0.15
Muslim/Sikh/Christian	19 (57.6%)	14 (42.4%)	
Education			
Illiterate or just literate	82 (70.7%)	34 (29.3%)	0.14
Educated up to intermediate	61 (70.1%)	26 (29.9%)	
Educated up to graduation or more	14 (51.8%)	13 (48.2%)	
Urban/Rural			
Urban	88 (76.5%)	27 (23.5%)	0.01*
Rural	69 (60%)	46 (40%)	
Type of Family			
Living alone	11 (73.3%)	4 (26.7%)	0.80
Nuclear family	112 (67.1%)	55 (32.9%)	
Joint family	34 (70.8%)	14 (29.2%)	
Diet Preference			
Vegetarian	77 (68.1%)	36 (31.9%)	0.97
Non-vegetarian	80 (68.4%)	37 (31.6%)	
Any Health Insurance			
Have health insurance	25 (64.1%)	14 (35.9%)	0.54
No health insurance	132 (69.1%)	59 (30.9%)	
Smoke			
Yes	41 (77.4%)	12 (22.6%)	0.10
No	116 (65.5%)	61 (34.5%)	
Diabetes			
Yes	99 (80.5%)	24 (19.5%)	0.01*
No	58 (54.2%)	49 (45.8%)	
Hypertension			
Yes	96 (75%)	32 (25%)	0.01*
No	61 (59.8%)	41 (40.2%)	
Sleep			
Regular	47 (57.3%)	35 (42.7%)	0.01*
Irregular	110 (74.3%)	38 (25.7%)	
Exercise			
Yes	52 (70.3%)	22 (29.7%)	0.6
No	105 (67.3%)	51 (32.7%)	

In this study, bivariate logistic regression was performed to identify variables that were potentially associated with depression, and those with a p-value less than 0.25 were selected for multivariate logistic regression analysis. The variables that were included in the final multivariable logistic regression model were gender, age category, religion, education status, place of residence, current smoking status, presence of hypertension and diabetes, and sleep habits. After adjusting for potential confounders, the results revealed that older age (greater than 80 years), rural residence, and non-diabetic status were associated with lower odds of depression. Specifically, individuals aged over 80 years had significantly lower odds of depression with an adjusted odds ratio (aOR) of 0.1 (95%CI: 0.1-0.4), p<0.01. Similarly, those residing in rural areas had lower odds of depression (aOR=0.3, 95%CI: 0.1-0.7, p<0.01) compared to their urban counterparts. Additionally, individuals without diabetes had lower odds of depression (aOR=0.2, 95%CI: 0.1-0.4, p<0.01) compared to those with diabetes. However, the multivariable logistic regression analysis did not reveal any statistically significant association between depression and the other variables studied, including gender, religion, education status, current smoking status, presence of hypertension, and sleep habits (Table 3).

**Table 3:** Multivariable logistic regression for factors associated with depression among the elderly age groups *= Statistically significant

Variables	Unadjusted odds ratio	p-value	Adjusted odds ratio	p-value
Gender				
Male	Reference		Reference	
Female	1.9 (1.1-3.4)	0.02*	1.6 (0.7-3.4)	0.02*
Age category				
60-69 years	Reference		Reference	
70-79 years	0.9 (0.5-1.8)	0.89	0.7 (0.3-1.5)	0.36
More than 80 years	0.4 (0.2-1.2)	0.11	0.1 (0.1-0.4)	0.01*
Religion				
Hindu	Reference		Reference	
Muslim/Sikh/Christian	0.6 (0.3-1.2)	0.15	0.8 (0.3-2.1)	0.72
Education				
Illiterate or just literate	Reference		Reference	
Educated up to intermediate	0.9 (0.5-1.8)	0.92	0.9 (0.4-2.2)	0.92
Educated up to graduated or more	0.4 (0.2-1.0)	0.06	0.8 (0.2-2.4)	0.64
Urban/Rural				
Urban	Reference		Reference	
Rural	0.5 (0.3-0.8)	0.01	0.3 (0.1-0.7)	0.01*
Smoke				
Yes	Reference		Reference	
No	0.6 (0.3-1.1)	0.18	0.6 (0.3-1.5)	0.32
Diabetes				
Yes	Reference		Reference	
No	0.3 (0.1-0.5)	0.01	0.2 (0.1-0.4)	0.01*
Hypertension				
Yes	Reference		Reference	
No	0.5 (0.3-0.8)	0.01	0.9 (0.4-1.9)	0.83
Sleep				
Regular	Reference		Reference	
Irregular	2.2 (1.8-3.8)	0.01	2.0 (0.9-4.1)	0.05

## Discussion

A cross-sectional community-based study was conducted to determine the prevalence of geriatric depression among 230 elderly individuals. The study also aimed to identify potential associations between depression and various factors such as age group, sex, marital status, education status, religion, place of residence, presence of co-morbidities, health insurance coverage, exercise habits, current smoking status, and sleeping habits of the participants. The overall prevalence of geriatric depression was found to be 68.2%. Univariate analysis demonstrated an association between depression and several factors such as sex, age group, education status, religion, place of residence, presence of comorbidities, exercise habits, smoking status, and sleeping habits. However, only four factors showed significant association with depression in multivariate logistic regression analysis, namely gender, age category, place of residence, and presence of diabetes.

The prevalence of depression among the study participants was 68.2%. The systematic review and meta-analysis conducted by Pilania et al. showed the prevalence of depression among elderly individuals in India to be 34.4% (95%CI: 29.3%-39.7%) [9]. However, the prevalence varied among different studies, with some showing a higher prevalence and others showing a lower prevalence. For example, a study conducted by Singh et al. reported a prevalence of 73.3% among elderly individuals selected from an old age home [12]. In contrast, a study conducted by Dhuria et al. in urban Delhi reported a prevalence of 45.6% [13]. The prevalence reported by Goswami et al.* *and Sahani et al. was 41.7% and 40.7%, respectively [14,15]. The differences in prevalence reported by these studies may be attributed to the different screening tools used. The studies conducted by Singh et al. and Dhuriaet al. used the Geriatric Depression Scale, whereas our study used the PHQ-9 questionnaire. Additionally, a study conducted in Uttarakhand using the PHQ-9 reported a prevalence of 11.2%; however, a different cut-off for depression was used [16]. The COVID-19 pandemic may also have contributed to the higher prevalence of depression among the elderly, as several studies suggest an increase in depressive episodes following the pandemic [17-19]. The elevated prevalence of depression witnessed in our study is of public health concern. Although the PHQ-9 questionnaire scores do not provide a definitive diagnosis of depression, this underscores the importance of conducting more in-depth evaluations to establish the presence of depressive disorders and to commence appropriate treatment. Early identification and intervention for depression, particularly among the elderly population, is necessary to improve their mental health and overall well-being.

The present study reveals a higher prevalence of depression in females (75%) compared to males (60.9%). This is consistent with previous epidemiological reports, which indicate that elderly females are more likely to be diagnosed with unipolar depression and report greater symptoms of depression than their male counterparts [14,20]. However, there are also studies that show the opposite trend, suggesting that depression is more common among males [7,21]. One study conducted by Paĺsson et al. examined the initial occurrence of depressive episodes in a longitudinal sample, which was tested every five years between the ages of 70 and 85. The results indicated that the incidence of initial depressive episodes increased with age for both men and women, but women were consistently more likely than men to experience an initial episode [22]. 

The present study revealed a higher prevalence of depression among individuals living in urban areas compared to those in rural areas. This finding is consistent with other studies conducted in India, which have also reported a higher prevalence of depression in urban areas [23]. A meta-analysis conducted by Purtle et al. highlights the potential impact of urbanization and population aging on the global burden of depression among older adults. The study emphasizes that the converging trends of urbanization and population aging may contribute to an increased burden of depression among older adults globally [24].

Our study observed a lower prevalence of depression in individuals aged over 80 years, in comparison to those within the age group of 60-69 years. However, studies conducted in India present a contrasting pattern, where the prevalence of depression tends to increase with age [25-27]. This inconsistency in results could be attributed to the proportion of individuals aged over 80 years in our study was only 6.9% of the total participants.

The prevalence of depression is known to be higher in individuals with diabetes. Specifically, research indicates that the prevalence of depression among individuals with diabetes is 80.5%, which is notably higher compared to the prevalence of depression among non-diabetics, which is 54.2%. These findings are consistent with studies conducted both in India and outside the country [28,29].

Depression among elderly individuals has emerged as a significant public health concern in recent years, owing to the decline in physical and physiological functioning associated with aging, along with changing family structures that contribute to a poorer quality of life. Although treatment options for depression have been shown to be equally effective in the elderly population as they are in younger individuals, depression among the elderly continues to be underdiagnosed and undertreated. In resource-limited healthcare settings, such as India, implementing depression care management strategies within primary care settings is crucial to improving outcomes for depressed elderly individuals [9]. This approach allows for the management of both physical comorbidities and depressive disorders simultaneously, leading to better overall clinical outcomes.

The study's significant contribution lies in its comparison of geriatric depression in elderly individuals living in urban and rural areas of Delhi, an area in Northern India. Despite the scarcity of such studies in this geographic region, this investigation sheds light on this important topic. Nevertheless, several limitations should be acknowledged. It should be noted that the screening method used in this study should not replace a clinician's diagnosis. Therefore, it is crucial to interpret the findings with caution. Additionally, the prevalence of depression was based on self-reported data, which may be susceptible to recall bias. Moreover, this study utilized a cross-sectional design, which limits the ability to establish causality.

## Conclusions

The current study highlights a high prevalence of depression amongst the elderly population. In particular, individuals who are female, living in urban areas, or have a history of diabetes were found to be significantly associated with depression. The results emphasize the importance of identifying and treating depression, particularly among vulnerable groups such as those living in urban areas and with pre-existing chronic conditions. Early detection through screening programs at the primary care level may be an effective strategy for managing depression in the elderly. 
